# Adaptation, acceptability and feasibility of Problem Management Plus (PM+) intervention to promote the mental health of young people living with HIV in Kenya: formative mixed-methods research

**DOI:** 10.1192/bjo.2022.564

**Published:** 2022-08-24

**Authors:** Moses Kachama Nyongesa, Eva Mwangome, Paul Mwangi, Carophine Nasambu, Judy Wanjiru Mbuthia, Hans M. Koot, Pim Cuijpers, Charles R. J. C. Newton, Amina Abubakar

**Affiliations:** KEMRI-Wellcome Trust Research Programme, Centre for Geographic Medicine Research (Coast), Kenya; and Department of Clinical, Neuro- and Developmental Psychology, Amsterdam Public Health Research Institute, Vrije Universiteit Amsterdam, The Netherlands; KEMRI-Wellcome Trust Research Programme, Centre for Geographic Medicine Research (Coast), Kenya; KEMRI-Wellcome Trust Research Programme, Centre for Geographic Medicine Research (Coast), Kenya; KEMRI-Wellcome Trust Research Programme, Centre for Geographic Medicine Research (Coast), Kenya; Uzima Mental Health Services, Kenya; Department of Clinical, Neuro- and Developmental Psychology, Amsterdam Public Health Research Institute, Vrije Universiteit Amsterdam, The Netherlands; Department of Clinical, Neuro- and Developmental Psychology, Amsterdam Public Health Research Institute, Vrije Universiteit Amsterdam, The Netherlands; KEMRI-Wellcome Trust Research Programme, Centre for Geographic Medicine Research (Coast), Kenya; Department of Public Health, Pwani University, Kenya; Department of Psychiatry, University of Oxford, UK; and Institute for Human Development, Aga Khan University, Kenya; KEMRI-Wellcome Trust Research Programme, Centre for Geographic Medicine Research (Coast), Kenya; Department of Public Health, Pwani University, Kenya; Department of Psychiatry, University of Oxford, UK; and Institute for Human Development, Aga Khan University, Kenya

**Keywords:** Common mental disorders, young people, HIV infections, Problem Management Plus, acceptability and feasibility

## Abstract

**Background:**

Problem Management Plus (PM+) is a psychological intervention that seeks to address common mental disorders among individuals exposed to adversity. Thus far, the potential for delivering PM+ by mobile phones has not been evaluated.

**Aims:**

To adapt PM+ for telephone delivery (ten weekly sessions of about 45 min each) and preliminarily evaluate its acceptability and feasibility with young people living with HIV (YLWH) in coastal Kenya.

**Method:**

This was a mixed-method formative research. Qualitative data collection included consultations with stakeholders, conducting key informant interviews with HIV care providers and focus group discussions with potential end-users, i.e. YLWH. Moreover, brief exit interviews with recipients of the adapted PM+ were conducted. Quantitative acceptability and feasibility indicators and outcome measures were tracked/assessed during PM+ preliminary implementation involving 70 YLWH.

**Results:**

From the qualitative inquiries, the adapted PM+ emerged as contextually appropriate, acceptable and feasible for mobile phone delivery, despite some concerns around missing nonverbal cues and poor network connectivity. High recruitment (85%) and fair programme retention (69%) were observed. Intervention sessions over the telephone lasted 46 min on average (range 42–55 min). Preliminary feasibility data indicated that the adapted PM+ has the potential of reducing common mental disorders among YLWH from the Kenyan coast.

**Conclusions:**

PM+ is acceptable and can feasibly be delivered via mobile phone to YLWH in coastal Kenya. This study sets the stage for a future fully powered, randomised controlled trial assessing the efficacy of the adapted PM+ in this or a similar setting.

Common mental disorders (CMDs), particularly depression and anxiety, are highly prevalent among people living with HIV (PLWH),^1^ including youths.^2^ Compared with an HIV-uninfected individual, the chances of an person with HIV developing a CMD are two to three times higher.^3^ Psychological disturbances in PLWH may result from psychosocial issues of living with HIV, such as HIV-related stigma,^4^ fear of premature death,^5^ poverty and financial strain in seeking HIV-related care, especially in resource-limited settings.^6^ The direct effects of HIV or its treatment, such as the neurologic effects of HIV on the brain^7^ and antiretroviral therapy (ART) side-effects,^8^ could also result in poor psychological well-being. CMDs among PLWH have been associated with negative outcomes like worsened prognosis of HIV infection,^9^ increased suicidality,^10^ ART nonadherence^11^ and poor quality of life.^12^

## An overview of interventions addressing CMDs in the context of HIV

Interventions seeking to address CMDs co-occurring with HIV, most based on the cognitive–behavioural therapy model, have proven to be acceptable, feasible and efficacious.^13,14^ However, most of the intervention work has been conducted in high-income countries of North America and Europe, with very little representation from resource-limited countries such as those in sub-Saharan Africa (SSA),^1,14^ where the majority of PLWH reside. Additionally, the target population for intervention research has been adults living with HIV, but not young people living with HIV (YLWH), who also experience comorbid mental health problems in large numbers.^2^ In SSA, interventions promoting the mental health of YLWH are notably scarce.^15^

The use of technology (computers or mobile phones) has shown to be a feasible delivery platform for mental health interventions among PLWH in high-income countries.^16^ The use of such technology, particularly mobile phones, for the delivery of mental health interventions has not been tested in the African context despite the rapidly increasing network connectivity and access to mobile phones.^17,18^ Compared with the conventional face-to-face intervention delivery, the advantages of telephone-based psychotherapy include flexible scheduling of sessions, use of client-preferred locations (e.g. because of concerns about stigma) and ease of service utilisation (e.g. no transportation costs incurred and reduced travel time).^19^ Such advantages may be particularly important for PLWH in resource-limited countries who cannot afford weekly transportation costs for attending physical therapy sessions because of financial difficulties (considering the already existing economic burden of regular HIV clinic attendance) or those with concerns about HIV-related and/or mental illness stigma.

## Study aims

This study describes the adaptation (contextualisation process) of a transdiagnostic multicomponent psychological intervention called Problem Management Plus (PM+), and preliminarily evaluates its acceptability and feasibility for mobile phone delivery. This work is purely formative and first efforts toward identifying alternative ways of addressing CMDs comorbid with HIV, more so in young people aged 18–24 years at the Kenyan coast. In SSA, we are not aware of any study that has evaluated PM+ in PLWH. However, PM+ has been tested among women exposed to gender-based violence in peri-urban settings of Kenya's capital, Nairobi.^20–22^

## Method

### Study design

This was a mixed-method formative research employing both qualitative and quantitative research methods.

### Study setting

The study was conducted between March 2019 and February 2020 in Kilifi and Mombasa Counties, coast of Kenya, through the Centre for Geographic Medicine Research-Coast (CGMR-C).

### Study participants and sampling

**Fig. 1:**
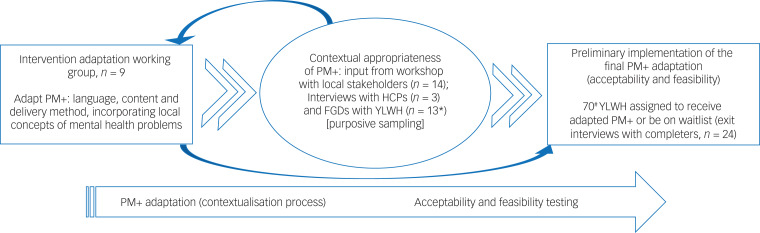
PM+ adaptation and preliminary implementation process. Workshop participants were stakeholders in HIV/mental health from Kilifi and Mombasa Counties. *These young people were identified from similar peer groupings at the HIV clinics to avoid accidental disclosure. ^#^These young people were identified and recruited from a larger cross-sectional study that was ongoing at that time recruiting from the HIV clinics in Kilifi and Mombasa Counties.^23^ FGD, focus group discussion; HCP, healthcare provider at the HIV clinic; PM+, Problem Management Plus; YLWH, young person living with HIV (aged 18–24 years).

Study participants were diverse, were selected using different sampling strategies and participated in different study activities, as shown in Figure 1.

### Overview of PM+ and the adaptation (contextualisation) process

PM+ was developed by the World Health Organization (WHO) to address the urgent need for affordable evidence-based mental health interventions for use in resource-limited settings, where the treatment gap for mental disorders is huge.^24^ It consists of four core intervention strategies taught by trained lay helpers in five weekly sessions lasting 90 min each. These strategies include managing stress through a slow breathing technique, a seven-step basic guide to managing practical problems, a behavioural activation strategy called ‘Get Going, Keep Doing’ and strengthening social support. In addition, a psychoeducation component is included in the first PM+ session, where individuals are taught about common reactions to adversity, including experiences of CMDs. Independent practice of PM+ strategies between sessions is encouraged to enhance learning through repetition. Action planning is also done to enhance the implementation of client-identified action points, and this is often reviewed in subsequent sessions.^24^

According to Chowdhary et al,^25^ the key elements of an intervention that require adaptation in a different cultural context include language, content and local conceptualisation of concepts (e.g. use of local idioms of distress or metaphors and appropriate illustrations/diagrams). All of these areas were considered during the adaptation of PM+, which involved a working group of three experienced translators, three senior field team members, a PM+ certified clinical psychologist and three researchers in the field of HIV and mental health. This working group held several intervention adaptation meetings for different purposes, some iterative in nature. First, the group aimed to harmonise translations (forward-translation to the local language, Swahili; back-translation to the original language, English) of all PM+ materials (intervention manual, interventionist guide and client handouts). Prior permission was sought from the WHO to translate all PM+ materials into Swahili. Second, the group critically evaluated PM+ in terms of content (strategies and accompanying activities, i.e. action planning) and delivery method (sessions, duration and delivery agent), and deliberated how best to adapt these for mobile phone delivery. Telephone therapy was identified *a priori* as a novel approach for addressing mental health treatment gap in the context of a highly stigmatised infection (HIV),^4^ and in the wake of rapidly increasing mobile phone access in Africa.^18^ Third, the group aimed to identify idioms of distress from the local literature and incorporate such in the adaptation of PM+, and to review PM+ illustrative materials for conceptual and contextual relevance.

The adapted version of PM+ by the working group was then tabled to relevant stakeholders and end-users (see Fig. 1) for discussions about its contextual appropriateness (in terms of content and delivery method) and areas for modifications. This process involved holding a workshop with stakeholders from both Kilifi and Mombasa Counties, three key informant interviews with healthcare providers from the HIV clinics and two focus group discussions with potential end-users (i.e. YLWH). Additionally, all participants that were engaged in refining PM+ adaptations were asked for their opinions about potential intervention implementation barriers and their suggestions for successful implementation of the final adaptation of PM+ in our setting among YLWH.

### Evaluation of the acceptability and feasibility of the adapted PM+: preliminary intervention implementation

Following incorporation of input from the discussions with stakeholders and potential end-users, the final adapted version of PM+ was preliminarily implemented in a small sample of 70 YLWH with mild-to-moderate symptoms of CMDs, identified and recruited within 2 weeks post-screening for CMDs among other interview questions administered in a larger cross-sectional study that was ongoing at that time.^23^ The goals were to evaluate the acceptability of the adapted PM+ by the intervention recipients (YLWH) and the feasibility of the proposed PM+ delivery methods, and preliminarily evaluate the feasibility of the adapted PM+ in promoting better mental health outcomes among YLWH with comorbid CMDs. Since this was purely formative research, no formal sample size and power computations were done. Sample size choice was informed by previous exploratory work.^21^

Participants were selected based on the following eligibility criteria: age 18–24 years and living with HIV, having mild or moderate symptoms of CMDs, consent for participation, access to a mobile phone (self/shared/borrowed), not at risk of committing suicide and not having an impairment owing to severe mental-neurological or substance use disorders.

**Fig. 2:**
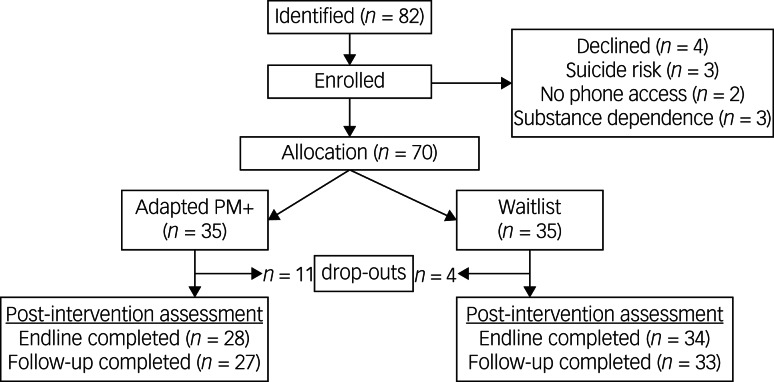
Participant flowchart. PM+, Problem Management Plus.

After providing written informed consent and doing baseline assessment (see section ‘Data collection procedure’), the 70 YLWH were randomly assigned to the intervention (*n* = 35) or waitlist (*n* = 35) groups by an independent statistician using computerised software. The study used electronic methods of data collection at all assessment time points (see section ‘Data collection procedure’). Figure 2 is the participant flowchart.

### Intervention conditions

#### PM+ group

These participants were linked to their PM+ helpers to get to know each other and to start the first face-to-face session. Toward the end of each session, the PM+ helper and the client scheduled a day and time for the subsequent phone session. At the end of the scheduled tenth session, brief intervention exit interviews were conducted with participants who completed all ten sessions. These participants also completed an anonymised intervention exit checklist.

#### Waitlist group

Waitlist participants received enhanced usual care (EUC). Given the national guidelines to screen for CMDs at HIV clinics as part of routine care of patients with HIV who are receiving treatment,^26^ EUC involved informing counsellors or healthcare providers at the HIV clinics (with participant permission) for further action as per the guidelines, but with no follow-up. Additionally, participants were informed to contact the study coordinator (contact details were provided to them) if they needed linking for psychological support. A 5–10 min weekly telephone call check-up was also made to waitlist participants to check their general well-being and to rule out suicidal risk/crises. Waitlist participants who completed follow-up assessments received the adapted PM+, although with substantial disruptions because of logistical challenges owing to the onset of COVID-19 in Kenya.

### Intervention delivery agent

The adapted PM+ was delivered by trained lay helpers who had completed at least high school education but did not have previous mental health training. These lay helpers were identified from the community through advertisement of the study position followed by an interviewing process, and were contracted through CGMR-C to implement the adapted PM+. None of the lay helpers had lived experiences as the young people they would provide care to, such as living with HIV and/or CMDs, except one who openly disclosed about living with HIV. A 3-week training programme (including 10 days of in-field practice) was delivered by a clinical psychologist (J.W.M.) trained on PM+, who was certified as a trainer of trainers and experienced with its previous implementation in Kenya.^20,21^ The apprenticeship model for training local providers was used.^27^ Four lay helpers (two females, two males; age range 26–35 years) and an additional two master-level staff at CGMR-C, who would provide supervision to the lay helpers, were trained. Training included an overview about CMDs, basic counselling skills, the tenets of the original PM+ intervention, the adaptations made and its proposed delivery. These helpers were also taught about self-care practices and psychological first aid principles.

### Competence and intervention fidelity

During training, competency was reinforced through multiple methods, including individual reading assignments, group activities and discussions, and role play with on-the-spot feedback. When preliminarily testing the adapted PM+, the lay helpers received weekly supervision to ensure adherence with the intervention structure and components. The supervisor formally observed at least three randomly selected sessions delivered by each PM+ lay helper, and provided feedback at the end. An observational rating tool, the Cognitive Therapy Adherence and Competence Scale (CTACS),^28^ was used. CTACS assesses both the quality of intervention delivery (i.e. competence) and adherence to the intervention structure (i.e. fidelity). Items are rated on a Likert scale of 0–6, with descriptive anchors 0 indicating ‘low competence/fidelity’, 2 indicating ‘some competence/fidelity’, 4 indicating ‘considerable competence/fidelity’ and 6 indicating ‘high competence/fidelity’. We only used 15 items of the CTACS, covering adherence and quality in the domains of intervention structure (nine items) and therapeutic relationship (six items).

For additional support, progress monitoring and brainstorming of arising issues, a physical meeting (lasting between 1.5 and 2 h) was held every fortnight between the PM+ helpers and the supervisors, with the trainer joining in via Skype call.

### Data collection procedure

Supplementary File 1 available at https://doi.org/10.1192/bjo.2022.564 summarises the data collection procedures during the PM+ adaptation process and preliminary implementation of the adapted version.

#### Qualitative data

During the PM+ adaptation process, qualitative data on its contextual appropriateness were collected through audio recording of discussions during the stakeholder workshop, key informant interviews and focus group discussions. Additionally, during the workshop, the Mentimeter Web 2.0 application (Mentimeter AB, Sweden; https://www.mentimeter.com/) was used to anonymously gather views from stakeholders on the overall acceptability of using the adapted PM+ to address the emotional and practical problems of YLWH in our setting. During preliminary implementation of the adapted PM+, qualitative data on its acceptability and feasibility were collected through brief intervention exit interviews with YLWH who completed all PM+ sessions. All discussions were guided by semi-structured interview guides.

#### Quantitative data

A nine-item intervention exit checklist based on ‘yes’ or ‘no’ response options was used to collect data from intervention recipients on the overall acceptability of the adapted PM+. Quantitative data on feasibility indicators of proposed PM+ delivery methods (i.e. recruitment and retention rate, session completion and duration) were tracked throughout the preliminary intervention implementation period and recorded on the respective client files. Data on participant recruitment and retention were electronically captured on Microsoft Excel (2019 for Windows®) spreadsheets. To evaluate the potential of the adapted PM+ in improving the mental health of YLWH with comorbid CMDs, the following locally validated mental health measurement tools were used as primary outcome measures: the nine-item Patient Health Questionnaire (PHQ-9)^29^ and the seven-item Generalised Anxiety Disorder Scale (GAD-7).^30^ For both the PHQ-9 and GAD-7, total scores of between 5–9 and 10–14 defined mild and moderate depressive or anxiety symptoms, respectively. Measures of secondary outcomes (quality of life and perceived social support) included the Functional Assessment of HIV Infection (FAHI) questionnaire, which has also been locally validated,^31^ and a Swahili version of the Social Provisions Scale (SPS).^32^ Total scores of the FAHI and SPS were analysed as continuous outcomes. All of the outcome measures were administered at baseline, endline (within 2 weeks after the conclusion of the tenth session) and follow-up (12 weeks after endline assessment) time points via audio computer-assisted self-interview.

### Ethics and consent statements

This study was performed in accordance with the ethical principles and guidelines for involving human participants as outlined in the Helsinki Declaration of 1975, revised in 2008. All study procedures were approved by the local institutional review board, the Scientific and Ethics Review Unit (SERU) of the Kenya Medical Research Institute (KEMRI) (approval number: KEMRI/SERU/CGMR-C/116/3632). All participants provided written informed consent for participation in this study. Participants received a token of appreciation (to compensate for their time and incurred travel costs) as per the guidelines in the approved study protocol.

### Data analysis

Qualitative data were analysed thematically with the assistance of NVIVO version 11 for Windows® (QSR International (Americas) Inc.; https://www.qsrinternational.com). All audio-taped materials were transcribed verbatim and uploaded to NVIVO for analysis as Microsoft Word (2019 for Windows®) files. Descriptive statistics such as frequencies, percentages and means were used to summarise the quantitative data on STATA version 15.0 for Windows® (StataCorp LP, College Station, Texas, USA). To identify the potential of the adapted PM+ in improving the mental health of YLWH in our setting, we checked the trend in mean scores on outcome measures of interest at the three assessment time points for both intervention and waitlist groups. We used the Student's *t*-test (independent and paired) to crudely compare the scores on outcome measures within and between the two groups.

## Results

### Sample characteristics

Table 1 summarises participant characteristics for both the qualitative and quantitative aspects of the study. For the quantitative aspect, the baseline participant characteristics are presented. There were no significant differences in all baseline characteristics of YLWH who received the adapted PM+ versus those on the waitlist.

**Table 1:** Participant characteristics

Characteristic	Qualitative aspect			Quantitative aspect		
	Workshop participant, *n* = 14	Key informants, *n* = 3	Focus group participants, *n* = 13	Intervention arm, *n* = 35	Waitlist arm, *n* = 35	*P*-value
Study site						
Kilifi	11	3	13	17 (48.6)	10 (28.6)	0.086
Mombasa	3	0	0	18 (51.4)	25 (71.4)	
Gender						
Male	5	0	6	13 (37.1)	11 (31.4)	0.615
Female	9	3	8	22 (62.9)	24 (68.6)	
Age (years)						
Range	29–58	36–50	18–24	18–24	18–24	
Mean (s.d.)	–	–	–	21.1 (2.4)	21.4 (1.8)	0.574
Education level						
Primary	0	0	3	18 (51.4)	12 (34.2)	
Secondary	0	0	8	12 (34.3)	15 (42.9)	
College/university degree	10	3	3	5 (14.3)	8 (22.9)	0.329
Postgraduate degree (Masters, PhD)	4	0	0	Not applicable	Not applicable	
Overall working experience						
<5 years	4	0	Not applicable	Not applicable	Not applicable	
5–10 years	2	1	Not applicable	Not applicable	Not applicable	
>10 years	8	2	Not applicable	Not applicable	Not applicable	
Viral load						
≤1000 copies/mL	Not applicable	Not applicable	Not applicable	24 (68.6)	20 (57.1)	0.322
>1000 copies/mL	Not applicable	Not applicable	Not applicable	11 (31.4)	15 (42.9)	
HIV clinical staging						
Stages 1 and 2	Not applicable	Not applicable	Not applicable	29 (82.9)	30 (85.7)	0.743
Stages 3 and 4	Not applicable	Not applicable	Not applicable	6 (17.1)	5 (14.3)	
ART duration						
>5 years	Not applicable	Not applicable	Not applicable	22 (62.9)	21 (60.0)	0.213
1–5 years	Not applicable	Not applicable	Not applicable	7 (20.0)	12 (34.3)	
<1 year	Not applicable	Not applicable	Not applicable	6 (17.1)	2 (5.7)	
BMI, kg/m^2^	Not applicable	Not applicable	Not applicable	19.7 (2.0)	21.0 (4.6)	0.149
Asset index score^a^	Not applicable	Not applicable	Not applicable	2.2 (1.8)	2.5 (1.5)	0.439

All data are in numbers (%) except age, which is a range or mean (s.d.). ART, antiretroviral therapy; BMI, body mass index.

a. Score range 0–7, higher scores indicate better socioeconomic status.

### Findings from the adaptation of PM+

All of the original PM+ materials in English language were adapted into Swahili. To enable mobile phone delivery, the working group proposed doubling the original PM+ intervention sessions (from five to ten weekly sessions) and reducing the session duration by half (from 90 min to approximately 45 min per session). The working group found the original PM+ strategies, illustrative materials (client hand-outs), accompanying activities (independent practice of sessions and action planning) and the use of lay helpers (as delivery agents) appropriate to the local context. Thus, when adapting PM+ for mobile phone delivery, these were retained as in the original PM+. The team also identified one study within the local context^33^ reporting on local idioms of distress such as ‘thinking too much’, ‘feeling sad’ and ‘restlessness’ and incorporated these idioms in the adapted PM+ materials. Supplementary File 2 provides a comparison between the original and adapted PM+. Supplementary File 3 shows the adapted ten session PM+ structure.

From the discussions of PM+ adaptations as proposed by the working group, workshop stakeholders, healthcare providers and YLWH found the content of the adapted PM+ culturally appropriate and endorsed the use of mobile phones for intervention delivery in the local context, using trained lay helpers (Table 2). Specifically, the use of mobile phones to deliver PM+ was lauded for enhancing confidentiality and boosting the client's confidence to open up and talk about issues, although a few participants raised the expected concern of missing nonverbal cues, such as this 29-year-old female workshop participant:

‘I agree that there is the beauty of opening up [with phone therapy] but then a con for me is I cannot see you, so your nonverbal cues you know, they are all lost. I have to guess from your tone whether you are angry, whether you are sad, I can't see these things!’

**Table 2:** Key themes and select illustrative quotes from the qualitative interviews on contextual appropriateness of PM+ adaptations

Theme	Illustrative quote
Cultural appropriateness of the proposed PM+ intervention strategies, and accompanying materials	‘Does it interfere with our culture in any way? I don't think. I think they [strategies] are appropriate.’ (Workshop participant, female, 36 years old) ‘Let's say you learn about the process of managing problems and then you understand well. When you encounter a problem, you can use the skills to find a solution to the problem.’ (Focus group discussion 1 participant, male, 21 years old)
Using mobile phones to deliver the adapted PM+	‘To me I will say it would enhance a lot of confidentiality in the sense that I am talking through the phone and I will decide where.’ (Key informant, female, 50 years old) ‘For me, I will say it is good because sitting to talk with someone is not easy. Some people are confident enough that they can sit and talk to someone about their problems, but there is also someone who can't. For this person, when you call, he/she now has the confidence of talking because they say aah! This person cannot see me, so let me open up and see whether they can help me.’ (Focus group discussion 1 participant, male, 21 years old)
Use of trained lay helpers^a^	‘To me, if somebody is trained and they have that knowledge and they have the competence, I think to me it's much okay.’ (Key informant, female, 36 years old) ‘I think it is a good idea to use trained lay helpers considering that also professionals are very busy to deliver that model [PM+]. And maybe these [lay helpers] are people who can even do a home visit and they are easily available.’ (Workshop participant, female, 32 years old) ‘To me it is okay because with a lay counsellor, it will be quick to open up and talk.’ (Focus group discussion 2 participant, female, 21 years old)
Delivering ten weekly intervention sessions of the adapted PM+	‘I think for me the sessions that I have seen with other interventions, ten weekly sessions are usually a good standard. I think for me it is adequate.’ (Workshop participant, female, 31 years) ‘Personally, I think it is okay [ten weekly sessions] because there is a place I come from and I cannot leave work and say I am coming here frequently.’ (Focus group discussion 2 participant, female 24 years old)
Delivering the adapted PM+ for 45 min per session	‘If it is done objectively, 45 min are enough because now I am thinking a scenario whereby, we are talking and there are issues to address, even it won't be enough.’ (Workshop participant, male, 33 years old) ‘That is okay, it is enough.’ Focus group discussion 1 participant, male, 23 years old)

PM+, Problem Management Plus.

a. None of the participants across the stakeholder workshop, key informant interviews and focus group discussions raised concern with the use of lay helpers for intervention delivery.

All participants supported the use of lay helpers as intervention delivery agents, citing reasons such as ease of their availability compared with mental health experts, being from the community and so having a better chance of clients establishing trust and opening up, and the fact that they would be trained (Table 2).

Participants found it contextually appropriate to deliver the adapted PM+ in ten weekly sessions of around 45 min each (Table 2). A 40-year-old female key informant said:

‘Ten weekly sessions is enough time for someone to be able to develop that trust and for the person that is handling these young people to be able to pick up the issues with a person.’

None of these participants suggested any modification to the content of adapted PM+ or its delivery method (i.e. ten weekly intervention sessions, each for around 45 min over the mobile phones, by trained lay helpers), but they provided some recommendations to ensure successful implementation of the intervention (Supplementary File 2). These recommendations were incorporated during the preliminary PM+ implementation phase.

### Acceptability and feasibility of the adapted PM+: preliminary implementation findings

#### Qualitative findings

Overall acceptability of the adapted PM+ was high from the anonymised Mentimeter inquiry during the stakeholder workshop (Supplementary File 4). During the exit interviews, YLWH who received the adapted PM+ reported that they found all of the taught strategies to be meaningful. Managing stress through the slow breathing technique was considered the most useful strategy by many of the YLWH. A 24-year-old female PM+ recipient said:

‘The strategy that helped me most was that of slow breathing. I used to be really stressed but now I am grateful. I was taught, I understood and began practicing what I was told.’

Use of lay helpers to implement the adapted PM+ was acceptable; none of the participants in the exit interviews raised any concern. A 22-year-old female recipient of PM+ said this during the exit interview:

‘My helper was good, he taught well, if you have not understood, he would explain again until you understand. I just thank him because I was in a bad situation that's why I even joined the programme, like this stress, I would keep to myself and remain in my room, but when the programme came, he has been of help to me.’

It is generally feasible to deliver the adapted PM+ over the telephone in ten weekly sessions of around 45 min each, according to the views from different study participants who received the adapted PM+. A 19-year-old male PM+ recipient said:

‘Ten weeks is good, like for those of us who are in school, you find sometimes you are held up you are busy, so one day-a-week session for 10 weeks is very good.’

Another 19-year-old male said:

‘For me, those 45 min were sufficient, we would talk and finish and even have some time for me to add anything.’

A 21-year-old male PM+ recipient said:

‘The time was not too long, not too short. It was the right time, although sometimes the lesson was so fantastic that you want it to extend.’

One participant who received the adapted PM+ (an 18-year-old male) suggested that for school-going YLWH, a 35 min session would be more appropriate to allow them plan for other school-related activities.

#### Quantitative findings

We recorded a recruitment (response) proportion of 85%. Twenty-four participants completed the intervention out of the 35 YLWH that were assigned to receive the adapted PM+ (69% retention rate). Of the 11 (31%) who dropped out, two had completed the first face-to-face PM+ session, six had completed between two and five sessions, and three had completed between seven and nine intervention sessions. On average, the sessions lasted for 46 min (range: 42–55 min). For the waitlist group, four YLWH dropped out. From the anonymised intervention exit checklist, overall acceptability of the adapted PM+ was high (Supplementary File 4).

PM+ delivery workload was evenly distributed among the four trained lay helpers. In terms of lay helpers’ competency to deliver the adapted PM+ and fidelity to the intervention structure, none of them scored zero in any of the items assessed with the CTACS; most scored ≥4 in many of the items, indicating good adherence. Items where a lay helper scored 2 were flagged for detailed feedback and discussion after the observed session.

Table 3 shows the trend in mean scores of primary and secondary outcome measures at baseline, endline and follow-up time points. At baseline, the intervention and waitlist groups did not differ in the mean scores on primary outcome measures of depressive (9.3 *v.* 10.0; *P =* 0.372) and anxiety (7.1 *v.* 7.8; *P =* 0.336) symptoms. At endline, a sharp and significant reduction in mean depressive (4.9 *v.* 8.9; *P =* 0.002) and anxiety symptom (4.6 *v.* 7.0; *P =* 0.030) scores was observed in the intervention compared with the waitlist group. Although the mean depressive and anxiety symptoms scores in the intervention group slightly increased at follow-up compared with endline time points, these within-group increases were not statistically significant (*P =* 0.275 and *P =* 0.626, respectively).

**Table 3:** Mean scores on outcome measure in intervention versus waitlist groups at baseline, endline and follow-up time points

	Intervention arm Mean (95% CI)	Waitlist arm Mean (95% CI)	*P*-value
Primary outcomes			
Depressive symptom scores (PHQ-9)			
Baseline	9.3 (8.2–10.4); *n* = 35	10.0 (8.8–11.3); *n* = 35	0.372
Endline (12 weeks)	4.9 (3.4–6.4); *n* = 28	8.9 (7.0–10.7); *n* = 34	**0**.**002**
Follow-up (24 weeks)	5.6 (3.5–7.6); *n* = 27	8.2 (6.5–10.0); *n* = 33	**0**.**044**
Anxiety symptom scores (GAD-7)			
Baseline	7.1 (6.0–8.2); *n* = 35	7.8 (6.8–8.8); *n* = 35	0.336
Endline (12 weeks)	4.6 (3.1–6.1); *n* = 28	7.0 (5.4–8.6); *n* = 34	**0**.**030**
Follow-up (24 weeks)	4.9 (3.3–6.4); *n* = 27	6.5 (5.1–7.9); *n* = 33	0.116
Secondary outcomes			
Perceived social support scores (SPS)			
Baseline	73.7 (69.9–77.6); *n* = 35	69.9 (65.5–73.7); *n* = 35	0.135
Endline (12 weeks)	76.3 (72.2–80.4); *n* = 28	71.1 (66.4–75.9); *n* = 34	0.103
Follow-up (24 weeks)	77.0 (71.6–82.4); *n* = 27	72.5 (67.9–77.1); *n* = 33	0.194
Quality of life scores (FAHI)			
Baseline	126.8 (117.2–136.4); *n* = 35	116.3 (107.0–125.7); *n* = 35	0.117
Endline (12 weeks)	137.5 (128.6–146.4); *n* = 28	123.0 (113.7–132.2); *n* = 34	**0**.**026**
Follow-up (24 weeks)	137.8 (127.1–148.5); *n* = 27	124.2 (113.3–135.1); *n* = 33	0.076

Statistically significant between-group differences are given in bold. For the PHQ-9 and GAD-7, lower scores indicate better mental well-being; for the SPS and FAHI, higher scores indicate better perceived social support and quality of life. PHQ-9, Patient Health Questionnaire (nine items); GAD-7, Generalised Anxiety Disorder Scale (seven items); SPS, Social Provisions Scale (24 items); FAHI, Functional Assessment of HIV Infection questionnaire (44 items).

For secondary outcomes, the intervention and waitlist groups did not differ in mean scores on measures of perceived social support (73.7 *v.* 69.9; *P* = 0.135) and quality of life (126.8 *v.* 116.3; *P =* 0.117) at baseline. At endline, a notable but insignificant increase in the mean social support scores was observed in the intervention compared with the waitlist group (76.3 *v.* 71.1; *P =* 0.103). The within-group mean social support scores slightly increased at follow-up for both the intervention and waitlist groups, but the increases were not statistically significant. For quality of life, a sharp and significant increase in the mean scores was observed in the intervention compared with the waitlist group (137.5 *v.* 123.0; *P* = 0.026) at endline. The mean quality-of-life score slightly increased at follow-up in both groups, but within-group increases in scores were statistically not significant.

### Intervention implementation barriers

In the stakeholder workshop, key informant interviews and focus group discussions, participants mentioned several barriers to anticipate and prepare for, mainly stemming from the use of mobile phones to deliver the intervention. The most common of these were the anticipated challenges of poor network coverage (especially in rural areas) and working with school-going YLWH (especially those in boarding schools where mobile phones are prohibited or when they are in class) and those without direct access to telephones (relying on a family member's mobile phone). A 37-year-old male workshop participant said:

‘For the school going, maybe the time you are calling them, they are in class. So that is the only challenge that I am foreseeing’

and

‘Another problem is network coverage or connectivity for somebody in the rural area; so, where there is no network connectivity, that is one of the few challenges I am foreseeing.’

Other identified barriers in the discussions with different participants included the challenges of setting up a session (calls going unanswered or the client being unreachable), young people's poor commitment to the intervention and mobile phones running out of charge during the session. A 40-year-old female key informant said:

‘And then another challenge with young people, it is not very easy to get them as required even if they promise you. They will promise you very well, but you will find that what they do is not just the way you agreed.’

Adequate preplanning with the client was recommended as a good strategy to help clients prepare in advance, hence avoiding some of the mentioned barriers, as explained by this 37-year-old male workshop participant:

‘I think there should be a pre-session call reminder a day before: “we will be having this call at this time” so that will make him or her [a YLWH] prepare in advance.’

During the preliminary implementation of the adapted PM+, some of the anticipated challenges never emerged as a barrier. For instance, no single session was disrupted because of a mobile phone running out of charge. Additionally, working with school-going YLWH was not as challenging as expected. With proper client–helper planning, sessions were delivered early in the morning, in the evening after classes or even during the weekend, as preferred by the client. However, in the exit interviews with some recipients of the adapted PM+, it emerged that the scheduled weekly sessions were sometimes disrupted because of the conflicting school schedule, as explained by this 18-year-old male participant:

‘It was challenging [having some sessions] because of the school programme, sometimes activities extended affecting the sessions in a way that we had to postpone some sessions.’

Indeed, some sessions were postponed to another time on the same session day or another day within the same week because of reasons such as participants not answering calls, being unreachable, reporting to be busy/engaged unexpectedly, external interruptions (e.g. from a family member) or not feeling well. As anticipated, poor network coverage also interrupted some session delivery, but this was addressed by retrying the phone call after some time and encouraging the client to find a confidential spot with better connectivity.

### Experiences of PM+

In the brief exit interviews with YLWH who received the adapted PM+, several noted positive changes in certain aspects of their lives. Some were able to handle stress better, like this 22-year-old female participant who said:

‘I used to be stressed, I couldn't do anything, and when stressed I locked myself [in the room] and cried. Now, I know how to control myself.’

Others were better placed to manage problems on their own, such as this 24-year-old female participant:

‘I am indeed very happy and thankful that PM+ came because many were suffering. Like myself, I didn't know what to do but now, I can manage my problems.’

Several other YLWH reported improvements in their interpersonal interactions (socialisation) with others, including family and friends. For instance, an 18-year-old male participant said:

‘I had a problem with social interaction and the sessions helped a lot because now I can mingle with others, I can talk to them; before I used to be alone in isolation.’

For a few others, their low mood reportedly improved, like this 24-year-old female participant:

‘I am grateful because I was so down and had given up, but working together with my helper, she encouraged me to practice the skills I learnt. I am grateful I can now move forward.’

One school going participant (an 18-year-old male) noted a positive change in attitude, saying:

‘I used to feel down because I was setting targets but not meeting them. Now, this really demoralized me to a point I didn't see value in education anymore. But now, there is a great change in that even if I don't reach my target, I know there is always a next time.’

Other participants reported that their ART adherence was much better now, like this 24-year-old female participant:

‘At first, I used to be irritated in regard to taking medication [antiretroviral]. I had given up. But after starting the training [PM+], my attitude changed, and my helper also encouraged me saying this [living with HIV] is not the end of the road. Now, I am doing well, I take my drugs well.’

## Discussion

### Summary of findings

This study describes a local adaptation of PM+ and evaluates its acceptability and feasibility among YLWH with comorbid CMDs at the Kenyan coast. To our knowledge, this is the first study that tests the acceptability and feasibility of implementing an adapted PM+ among the HIV-infected population, specifically young people. Findings reveal that adaptation of PM+ for mobile phone delivery – in ten weekly sessions of around 45 min each, by trained lay helpers – is contextually appropriate in settings such as the Kenyan coast. Preliminary feasibility data indicate that the adapted PM+ has the potential of improving mental health outcomes in YLWH.

### Comparison with findings from other studies

This formative study from SSA is the first to look at the acceptability of delivering strategies of a psychological intervention through mobile phones. Other psychological interventions in this setting have been delivered through the conventional face-to-face approach.^13^ Acceptability of mobile phone use for PM+ delivery was high based on the discussions with different participants, although a few expressed reservations such as the obvious difficulty of picking nonverbal cues. Nonetheless, the acceptability of using mobile phones sets the stage for thinking about alternative ways of offering evidence-based psychological interventions in SSA, considering that in this setting, the majority of the population are increasingly owning these gadgets (mostly basic mobile phones).^18^ In SSA, telephone therapy can also offer a long-lasting solution to accessing psychological support as far as the two highly stigmatised conditions are concerned, i.e. HIV and mental illness. Telephone therapy sessions can also continue uninterrupted during disease outbreaks such as the ongoing COVID-19, which requires stringent safety measures, including social distancing and movement restrictions.

In resource-limited settings, the use of trained lay counsellors for the delivery of psychological interventions has been preferred because of the shortage of mental health experts.^34^ Interventions such as the Friendship Bench project in Zimbabwe^35^ have shown how important trained lay counsellors are in offering psychological support. The high acceptability and successful preliminary implementation of the adapted PM+ by lay helpers in this study suggests that lay helpers can adequately deliver PM+ after thorough training and under continued supervision. The use of lay helpers was also acceptable in another PM+ exploratory study in Kenya.^21^

The high recruitment of participants into the study supports the feasibility of enrolling individuals with HIV into the PM+ programme. We observed a fair intervention retention of 69%, which almost compares to the retention of 73% observed in a pilot study delivering PM+ through the conventional face-to-face approach.^36^ One reason that may have contributed to participant attrition was the lack of direct access to a mobile phone. Some participants who borrowed a mobile phone for the sessions opted out of the programme, the main reason being avoidance of too much questioning around borrowing a phone on a weekly basis and using it for close to an hour. For YLWH struggling with mobile phone accessibility, support for the acquisition of basic types of mobile phones may increase their retention in the intervention programme, but there must first be an assessment of return on investment.

In this study, discussions with different stakeholders revealed that all retained PM+ intervention strategies were contextually appropriate. When implemented, we observed a trend toward a reduction in CMD symptom scores at endline assessment, which was significantly lower among YLWH who received PM+ compared with those on the waitlist despite the study not being powered to detect meaningful differences. Similar findings have been reported in PM+ pilot studies involving women facing gender-based violence in Nairobi, Kenya,^21^ and individuals affected by conflict in Peshawar, Pakistan.^36^ Regardless of the delivery platform, PM+ strategies appear to improve the mental health of individuals experiencing different kinds of adversities. This proposition is supported by the positive experiences reported by participants in this study and another qualitative enquiry.^22^

Several barriers can interrupt the delivery of PM+ sessions over the mobile phone. Some of these barriers were beyond the control of the helpers, such as conflicting school schedules and clients not answering calls or being unreachable over the telephone, and necessitated postponement of sessions. Others, such as poor network connectivity, were addressable barriers. With adequate client preparation during the pre-session reminder calls, some anticipated challenges such as mobile phones running out of charge during sessions were averted. These barriers expose what would be expected in the real-life scale-up of an intervention model like the one we propose. Potential ways of working around some of the experienced barriers include encouraging clients to have telephone sessions in places with known good network coverage, having alternative contact details for tracing unreachable clients and constantly reminding clients of the importance of regular session attendance.

### Interpretation of study findings and implications for future work

From the findings of this study, the adapted PM+ appears contextually appropriate within the coastal Kenyan setting. The use of mobile phones to deliver PM+ is highly acceptable and so is the use of lay helpers. In this setting, it is feasible to recruit and retain YLWH in the adapted PM+ programme, as an excellent response rate and fair retention rate were observed. It is also feasible to implement ten weekly PM+ sessions of around 45 min each via mobile phones. Preliminary feasibility data indicates that PM+ has the potential to reduce CMDs in YLWH. The findings from this study inform and justify a fully powered, definitive, randomised controlled trial in the future, to assess the efficacy of the adapted PM+ with the HIV-infected population living in the Kenyan coast or a similar setting. There may be need to assess further the influence of continued therapy on sustaining positive effects/desired outcomes, beyond the indicated ten weekly sessions. Additionally, some of the positive experiences reported by YLWH who received the adapted PM+ indicate the need to evaluate further PM+ effect on reducing HIV-related general psychological distress and improving HIV treatment outcomes of YLWH, specifically ART adherence.

### Study limitations

We acknowledge some limitations that should be considered when interpreting the findings from this study. First is the sample size. Because of time constraints, we only conducted a few key informant interviews and focus group discussions, largely in Kilifi County. Relatedly, although we preliminarily detected significant differences by the intervention condition, sample size and power calculations were not done to detect such differences during the design of the study, as this was considered purely formative work. We deliberately did not include the acceptability and feasibility views of PM+ helpers because we anticipated bias (lay helpers giving socially desirable answers), as they were contracted and salaried by CGMR-C, through which the PM+ programme was being run. Lastly, we did not ask study participants about how telephone therapy sessions at home affected factors such as HIV-related stigma and unintentional HIV status disclosure, as part of the implementation feedback.

## Data Availability

The data that support the findings of this study can be requested through a written application to the Data Governance Committee (DGC) of the KEMRI-Wellcome Trust Research Programme, using the email dgc@kemri-wellcome.org. The DGC will review the application and advise as appropriate, ensuring that uses are compatible with the consent obtained from participants during data collection. The data is not publicly available as some of it contains private and confidential information collected from a special population of individuals living with HIV.

## Supplementary material

For supplementary material accompanying this paper visit http://doi.org/10.1192/bjo.2022.564. click here to view supplementary material
