# Supplementary material for: Adaptation, acceptability and feasibility of Problem Management Plus (PM+) intervention to promote the mental health of young people living with HIV in Kenya: formative mixed-methods research
Source: BJPsych Open. 2022 Aug 24;8(5):e161. doi: 10.1192/bjo.2022.564 (PMC9438483; doi:10.1192/bjo.2022.564)
Supplement: Supplementary file 1 [file S2056472422005646sup001.zip › S2056472422005646sup001/Suppl. file 1 data collection procedures.pdf]

Supplementary file 1: Data collection procedures

Evaluation aspect	Data collection procedures
Acceptability and cultural appropriateness of proposed PM+ intervention strategies or components, and accompanying activities	• Workshop with stakeholders<br>• Key informant interviews with HIV care providers<br>• Focus group discussions with potential end-users<br>• Exit interviews with intervention recipients
Use of mobile phones to deliver the adapted PM+: Contextual appropriateness and acceptability by end-users	• Workshop with stakeholders<br>• Key informant interviews with HIV care providers<br>• Focus group discussions with potential end-users<br>• Exit interviews with intervention recipients
Use of trained lay helpers: Contextual appropriateness and acceptability by end-users	• Workshop with stakeholders<br>• Key informant interviews with HIV care providers<br>• Focus group discussions with potential end-users<br>• Exit interviews with intervention recipients
Overall acceptability of adapted PM+	• Workshop with stakeholders (anonymous mentimeter.com)<br>• Intervention exit checklist (anonymous)
Delivering 10 weekly intervention sessions of the adapted PM+: Contextual appropriateness and feasibility	• Workshop with stakeholders<br>• Key informant interviews with HIV care providers<br>• Focus group discussions with potential end-users<br>• Exit interviews with intervention recipients<br>• Tracking of indicators during PM+ implementation (sessions completed)
Delivering the adapted PM+ for ~45 minutes per session: Contextual appropriateness and feasibility	• Workshop with stakeholders<br>• Key informant interviews with HIV care providers<br>• Focus group discussions with potential end-users<br>• Exit interviews with intervention recipients<br>• Tracking of indicators during PM+ implementation (session duration)
Feasibility of recruiting and retaining participants	• Tracking of indicators during PM+ implementation (recruitment, retention, and dropout rate)
Feasibility of the adapted PM+ in addressing CMDs	• Tracking of indicators during PM+ implementation (trends in outcome scores at different time points)
