# Supplementary material for: Adaptation, acceptability and feasibility of Problem Management Plus (PM+) intervention to promote the mental health of young people living with HIV in Kenya: formative mixed-methods research
Source: BJPsych Open. 2022 Aug 24;8(5):e161. doi: 10.1192/bjo.2022.564 (PMC9438483; doi:10.1192/bjo.2022.564)
Supplement: Supplementary file 1 [file S2056472422005646sup001.zip › S2056472422005646sup001/Suppl. file 2 original vs adapted PM+ and suggestions for successful implementation.pdf]

Supplementary file 2: A comparison of the original versus adapted PM+ and participant suggestions for successful implementation of the adapted PM+

A comparison between the original and adapted PM+

Component	Original PM+	Adapted PM+
Language	English	Swahili
Intervention strategies<br>(CBT-based)	Psychoeducation<br>Managing stress<br>Managing problems<br>Behavioural activation<br>Strengthening social support	Psychoeducation<br>Managing stress<br>Managing problems<br>Behavioural activation<br>Strengthening social support
Number of sessions	5-weekly sessions	10 weekly sessions
Duration of session	90 minutes	~45 minutes
Mode of delivery	Face-to-face sessions	Phone call sessions* (phone therapy) except the first session which is face to face (getting to know each other)
Interventionist	Trained lay helpers/providers	Trained lay helpers/providers
**Note. *** Participants needed to have access to a phone whether self-owned, shared or borrowed		

Suggestions from the interviews with participants for making the implementation of the adapted PM+ a success

Recommendation	What was done
• Use of pocket friendly client handouts than A4 size	We redesigned the supporting worksheets for clients into pocket size pamphlets.
• Using young PM+ providers for easy connection with YLWH	We identified, recruited, and trained youthful PM+ helpers 35 years or below
• Need to have an open and honest communication to establish trust while working with young people	These virtues were emphasized during the training of the PM+ helpers and throughout the project implementation
• Flexibility with the 45-minute phone therapy; some sessions will spill over; others will be short	While an emphasis was placed on being objective during sessions, we encouraged the PM+ helpers to be open-minded and not overly concerned with having to finish the session at exactly 45 minutes
• Reminder calls a day before the scheduled session day	Reminder calls were made, also encouraging the client to prepare for the upcoming session e.g. charging phones, taking sessions in an area with good network coverage
