# Supplementary material for: Adaptation, acceptability and feasibility of Problem Management Plus (PM+) intervention to promote the mental health of young people living with HIV in Kenya: formative mixed-methods research
Source: BJPsych Open. 2022 Aug 24;8(5):e161. doi: 10.1192/bjo.2022.564 (PMC9438483; doi:10.1192/bjo.2022.564)
Supplement: Supplementary file 1 [file S2056472422005646sup001.zip › S2056472422005646sup001/Suppl. file 4 overall acceptability of the adapted PM+ .pdf]

Supplementary file 4: Overall acceptability of the adapted PM+ from anonymous inquiry

When workshop participants were anonymously asked for their general opinion on using the adapted PM+ to address emotional and practical problems of YLWH in our setting, acceptability was high as shown in **Suppl**. **file 4, Fig. 1** below.

Suppl. file 4, Fig. 1: Stakeholder opinion on using the adapted PM+ to address emotional and practical problems of YLWH at the Kenyan coast, n=14

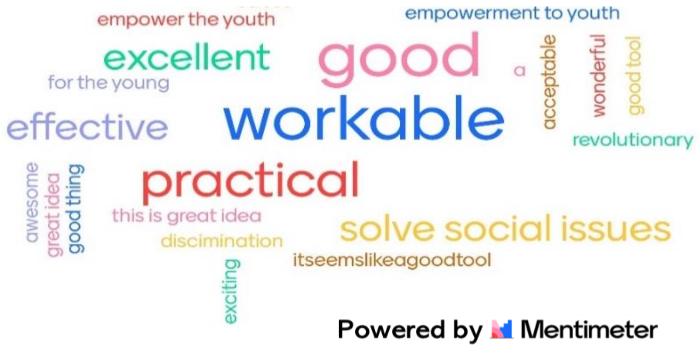

One participant mentioned that the proposed approach was discriminatory, a comment we think was related to the perception that the intervention was only going to target YLWH owning a mobile phone. We had to clarify to the audience that YLWH who do not own a mobile phone but can access one during the weekly session, were also eligible for participation.

From the intervention exit checklist that was anonymously completed by YLWH, acceptability of PM+ was also high as shown in **Suppl. file 4, Table 1** below.

Suppl. file 4, Table 1: Anonymous intervention exit checklist feedback, n=24

Item	Yes	No
I enjoyed the training	24 (100%)	-
I felt satisfied after training	24 (100%)	-
I felt tired after the training	2 (8.3%)	22 (91.7%)
The strategies taught were easy to understand	24 (100%)	-
The client appendix was an easy reference during teaching of strategies	23 (95.8%)	1 (4.2%)
The program easily fit in my weekly schedule	22 (91.7%)	2 (8.3%)
The training felt therapeutic to me	23 (95.8%)	1 (4.2%)
The training felt useless to me	-	24 (100%)
I would use this training outside of this study e.g. in personal challenges, teach others etc	23 (95.8%)	1 (4.2%)

When asked whether they can recommend PM+ to be given to other YLWH facing emotional or practical problems, all the recipients of the adapted PM+ gave strong positive recommendations during the exit interviews.

"I recommend this training [PM+], because it is good, it is taught well, and can move one from one step to another."
(Exit interview participant, Female, 24 years)
