# Supplementary figures and images for: Adaptation, acceptability and feasibility of Problem Management Plus (PM+) intervention to promote the mental health of young people living with HIV in Kenya: formative mixed-methods research
Source: BJPsych Open. 2022 Aug 24;8(5):e161. doi: 10.1192/bjo.2022.564 (PMC9438483; doi:10.1192/bjo.2022.564)

### Supplementary file 3: The adapted 10 session PM+ structure

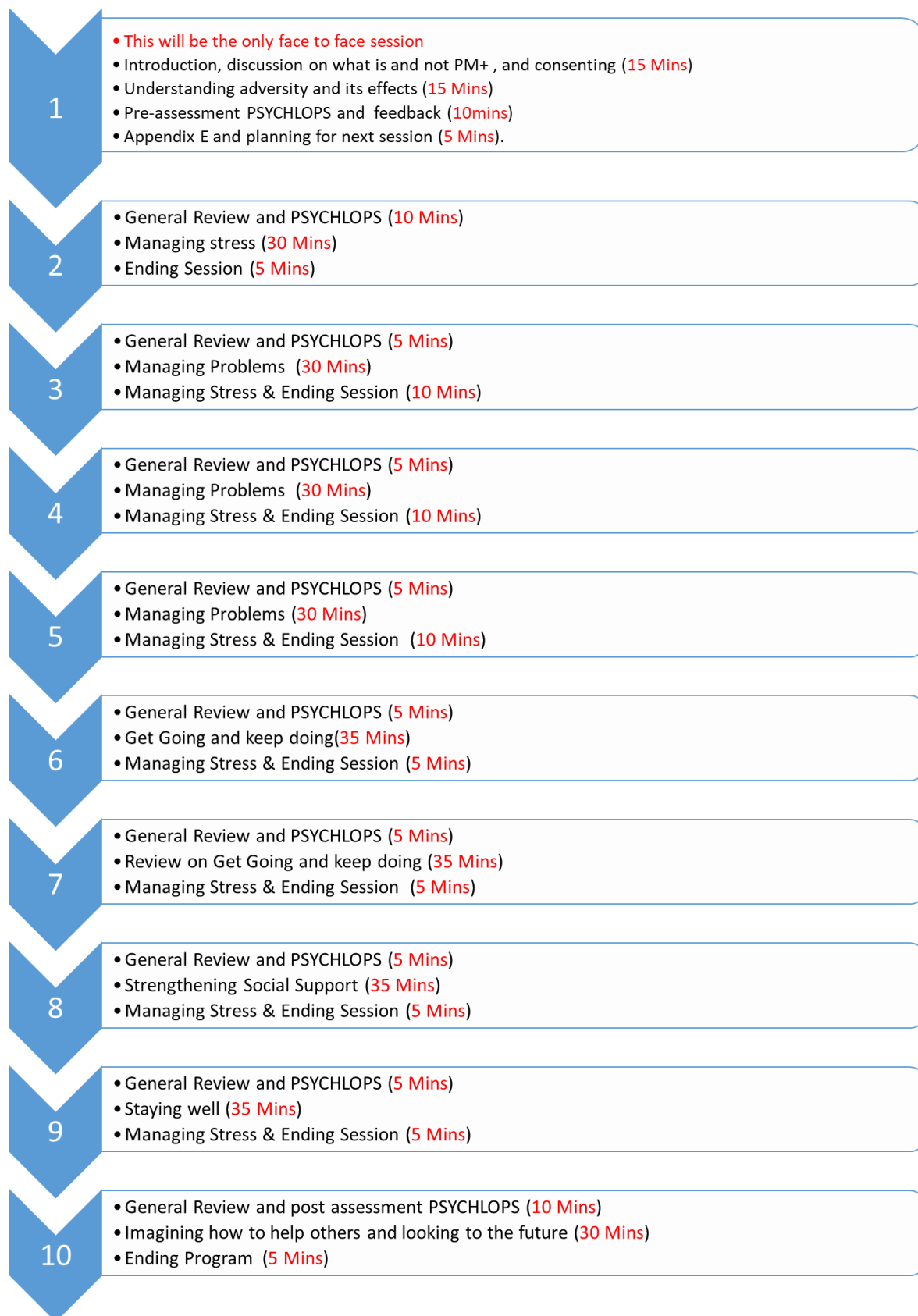

Supplement: Supplementary file 1 [file S2056472422005646sup001.zip › S2056472422005646sup001/Suppl. file 3 the adapted 10 session PM+ structure.pdf]
